# Fumarate hydratase c.914T > C (p.Phe305Ser) is a pathogenic variant associated with hereditary leiomyomatosis and renal cell cancer syndrome

**DOI:** 10.1002/mgg3.1293

**Published:** 2020-05-28

**Authors:** Kelsey E. Breen, Maria I. Carlo, Yelena Kemel, Anna Maio, Ying‐Bei Chen, Liying Zhang, Ozge Ceyhan‐Birsoy, Diana Mandelker

**Affiliations:** ^1^ Department of Medicine Memorial Sloan Kettering Cancer Center New York City NY USA; ^2^ Niehaus Center for Inherited Cancer Genomics Sloan Kettering Institute Memorial Sloan Kettering Cancer Center New York City NY USA; ^3^ Department of Pathology Memorial Sloan Kettering Cancer Center New York City NY USA; ^4^ Department of Pathology and Laboratory Medicine University of California Los Angeles CA USA

**Keywords:** carcinoma, fumarate hydratase, leiomyomatosis, mutation, renal cell

## Abstract

**Background:**

Hereditary leiomyomatosis and renal cell cancer syndrome (HLRCC), caused by heterozygous germline pathogenic variants in the *FH*, confers an increased risk for cutaneous and uterine leiomyomas and renal cancer.

**Methods:**

About 13,722 advanced cancer patients, including 560 with renal cell carcinoma, had germline analysis performed in the context of tumor‐normal sequencing under an IRB approved protocol.

**Results:**

We report two unrelated individuals with early onset kidney cancer who both carried the c.914C > T (p.Phe305Ser) germline variant in the *FH*. Both tumors exhibited loss of FH staining by immunohistochemistry and/or positive 2SC staining. Subsequent familial testing discovered that a daughter of a proband who carried the variant had both cutaneous and uterine leiomyomas.

**Conclusion:**

This combination of evidence suggests that the *FH* c.914C > T (p.Phe305Ser) is pathogenic for HLRCC.

## Graphical abstract

Immunohistochemical analysis of kidney tumors from two unrelated patients with the germline FH c.914T > C (p.Phe305Ser) variant revealed a loss of fumarate hydratase (FH) staining and positive S‐(2‐succino)‐cysteine (2SC) staining. We provide evidence to suggest the FH variant c.914C > T (p.Phe305Ser) is pathogenic for HLRCC.

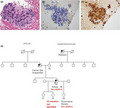

## 1 INTRODUCTION

Hereditary leiomyomatosis and renal cell cancer (HLRCC) is an autosomal dominant cancer predisposition syndrome caused by heterozygous germline pathogenic variants in the fumarate hydratase (*FH;* OMIM*136850) gene (Tomlinson et al., 2002). HLRCC is characterized by uterine leiomyomas, cutaneous leiomyomas, and renal cell carcinoma (RCC) (Launonen et al., 2001; Lehtonen et al., 2006; Reed, Walker, Walker, & Horowitz, 1973; Stewart et al., 2008). While the majority of individuals with HLRCC exhibit cutaneous leiomyomas and almost all females with HLRCC present with uterine leiomyomas, the penetrance of RCC is variable but may be as high as 19% (Muller et al., 2017; Skala, Dhanasekaran, Dhanasekaran, & Mehra, 2018).

HLRCC‐associated kidney cancers were initially recognized by their unique histologic features; however, a spectrum of growth patterns has been demonstrated and recent classifications have recognized FH deficiency as a distinct entity. Biallelic inactivation of FH results in fumarase deficiency, causing increased levels of intracellular fumarate and subsequent production of S‐(2‐succino)‐cysteine (2SC) (Toro et al., 2003; Wei et al., 2006). Positive nuclear and cytoplasmic 2SC staining and a loss of FH staining via immunohistochemistry have been shown to be highly specific in identifying HLRCC‐associated renal tumors (Bardella et al., 2011; Chen et al., 2014; Trpkov et al., 2016). The detection of FH deficiency by immunohistochemistry in a significant proportion of tumors histologically diagnosed as “unclassified RCC” or “papillary RCC type 2” demonstrates the need to establish immunohistochemistry‐based screening strategies for these tumors (Chen et al., 2014; Gupta et al., 2019; Trpkov et al., 2016).

Individuals with FH‐deficient tumors are routinely referred for further genetic evaluation. HLRCC‐associated renal tumors are typically early onset (Bhola, Gilpin, Gilpin, Smith, & Graham, 2018; Menko et al., 2014), more aggressive, and with a higher risk to metastasize (Grubb et al., 2007; Trpkov et al., 2016). Establishing a molecular diagnosis of HLRCC is critical for identifying at‐risk family members to offer predictive genetic testing and facilitate preventive screening measures. While there is not a clear consensus on surveillance recommendations for HLRCC, recent guidelines propose that individuals undergo annual magnetic resonance imaging (MRI) of the abdomen, annual dermatologic exam, and annual gynecologic exam for females (Carlo et al., 2019; Pithukpakorn & Toro, 1993; Schultz et al., 2017).

Given that HLRCC is a rare syndrome, it is important to appropriately classify variants within the *FH* to facilitate an accurate diagnosis. Here, we describe two cases of HLRCC with a novel germline heterozygous *FH* variant. We provide immunohistochemical and segregation evidence toward pathogenicity of this specific *FH* variant. These findings may be important in reclassifying this variant across genetic testing laboratories.

## 2 METHODS

### 2.1 Ethical compliance

Individuals in this study consented to a Institutional Review Board (IRB)‐approved protocol at Memorial Sloan Kettering Cancer Center.

### 2.2 Cases

The two individuals with the *FH* germline c.914C > T (p.Phe305Ser) variant were identified from the cohort of patients profiled using the Memorial Sloan Kettering Mutation Profiling of Actionable Cancer Targets (MSK‐IMPACT) assay (*n* = 13,722) who consented to germline analysis under an Institutional Review Board (IRB)‐approved protocol at MSKCC. Somatic analysis was performed on 410 or 468 genes, depending on the version of the assay, and germline analysis was performed on 76 or 88 genes, respectively (Mandelker et al., 2017). Sequencing and variant calling were performed as described previously (Cheng et al., 2015). Germline variants on the 76 or 88 gene panel were reviewed by board‐certified molecular pathologist or clinical molecular genetics and classified based on the American College of Medical Genetics (ACMG) criteria (Richards et al., 2015).

### 2.3 Immunohistochemistry

Immunohistochemical staining for FH was performed using a mouse monoclonal antibody (clone J‐13, Santa Cruz Biotechnology) as previously described (Smith et al., 2016). An absence of FH staining in the neoplastic cells, in the presence of positive internal control (cytoplasmic, granular staining in non‐neoplastic cells), was interpreted as lost or FH‐deficient status. Immunohistochemical staining for S‐(2‐succino)‐cysteine (2SC) was performed using a polyclonal antibody as described previously (Bardella et al., 2011; Chen et al., 2014). Briefly, 4‐*μ*m thick sections from representative formalin‐fixed, paraffin‐embedded tissue blocks were processed using the Ventana Discovery XT system with antigen retrieval (CC1 solution, 60 min), primary antibody (1:2,000), and OptiView DAB IHC detection steps (Ventana). The presence of diffuse, nuclear, and cytoplasmic staining was interpreted as positive.

## 3 RESULTS

### 3.1 Case #1

**FIGURE 1:**
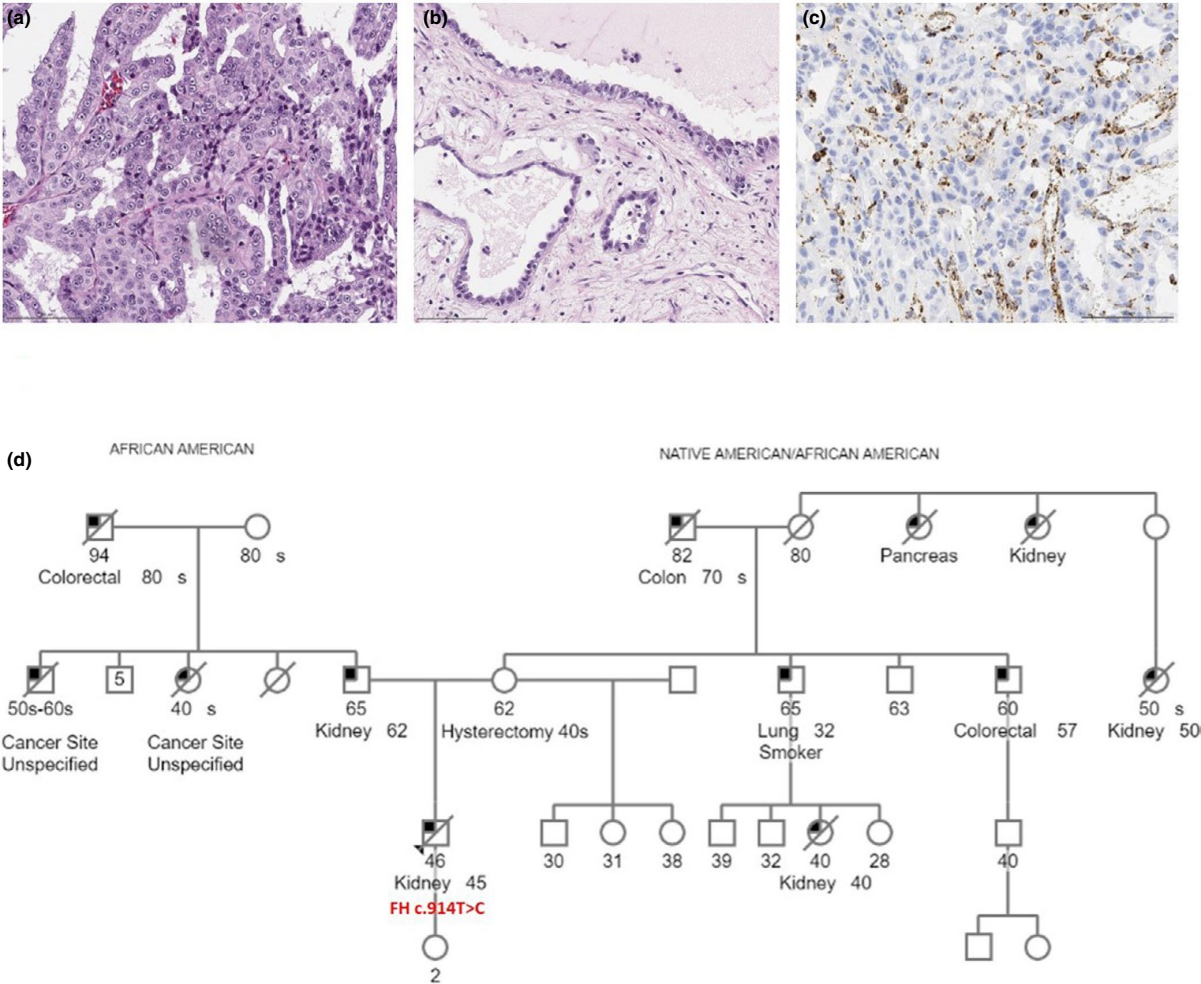
Immunohistochemical analysis and pedigree for case #1. Representative images of the patient's kidney tumor show (a) papillary and (b) tubulocystic areas. Tumor cells show prominent nucleoli and perinucleolar halos. (c) immunohistochemical analysis of the kidney tumor revealed a loss of fumarate hydratase (FH) staining. (d) The proband (indicated with the arrow head) was a 46‐year‐old man who was diagnosed with kidney cancer at the age of 45. His maternal family is notable for early onset kidney cancer, although no one else in the family has pursued genetic testing to date

A 45‐year‐old male without a reported significant medical history presented with gastrointestinal reflux and indigestion to his gastroenterologist, who palpated a large left abdominal mass. Computed tomography (CT) of the abdomen and lung revealed a left renal mass with retroperitoneal adenopathy and pulmonary lesions suspicious for metastatic disease. He underwent left radical nephrectomy and regional lymphadenectomy. Pathology revealed a 13.6‐cm renal mass extending into renal sinus and perirenal fat, invading renal vein, and with metastasis to the adrenal gland and 5/5 lymph nodes. The tumor displayed papillary, tubulocystic, and infiltrating tubular and solid growth patterns and showed prominent nucleoli and perinucleolar halos that were highly suspicious for FH‐deficient renal cell carcinoma (Figure 1a and b). The diagnosis was confirmed by a loss of FH protein expression in tumor cells (Figure 1c). The patient was referred to clinical genetics for further evaluation, where his personal and family histories were elicited by a genetic counselor. The patient's maternal family was significant for: his mother who had a hysterectomy in her 40s for uterine fibroids, a first cousin who was diagnosed with clear cell kidney cancer at age 40 and died of the disease at age 40, his grandmother's sister who also died of kidney cancer at an unknown age, and his grandmother's niece (the patient's first cousin once removed) who died in her 50s with kidney cancer. The patient's paternal family history is significant for his father who was diagnosed with kidney cancer at age 62. Little information was available pertaining to this diagnosis. The patient is of African American and Native American descent (Figure 1d). In light of the patient's FH‐deficient kidney tumor and significant family history, the patient was referred for a dermatologic evaluation. Complete cutaneous examination was performed without documentation of any cutaneous leiomyomas. During the clinical genetics consultation, he provided a blood sample for full sequencing and large rearrangement analysis of the *FH*. An outside laboratory reported the c.914T > C (p.Phe305Ser) variant, and classified it as a variant of uncertain clinical significance. Next‐generation sequencing (NGS) analysis of the patient's renal tumor revealed the following heterozygous somatic alterations: *FH* (NM_000143 ‐ 1q43) intragenic deletion involving the loss of exon 1–2, *KDM5C* (NM_004187) exon 7 p.K289E (c.865A > G), and *NUF2* (NM_031423) exon 8 p.S171C (c.512C > G). Germline analysis of 88 cancer predisposition genes only revealed the known heterozygous c.914T > C (p.Phe305Ser) variant in the *FH*. The patient received systemic treatment with progression of disease and died 14 months from diagnosis.

### 3.2 Case #2

**FIGURE 2:**
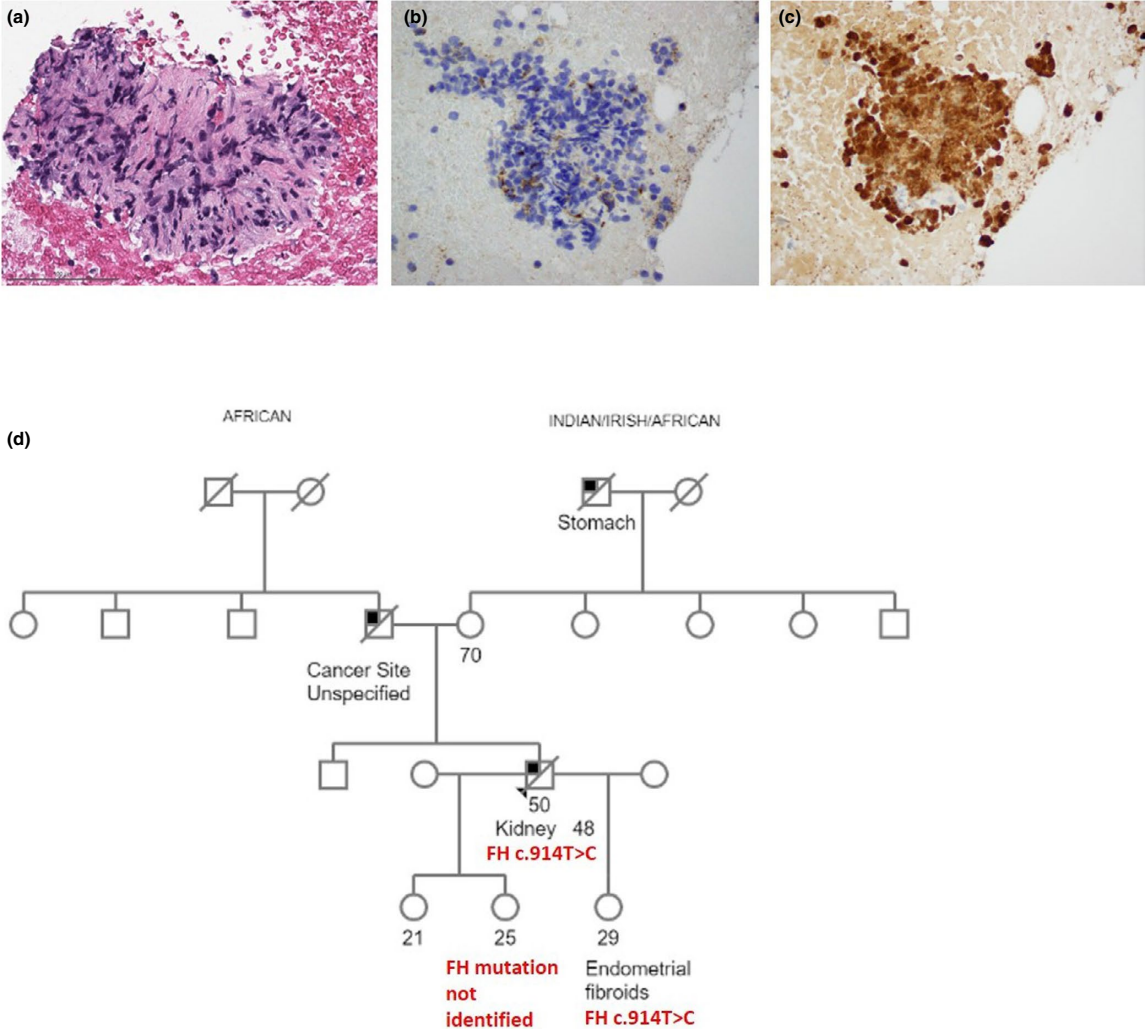
Immunohistochemical analysis and pedigree for case #2. (a) Bone biopsy reveals partially crushed epithelioid and spindled neoplastic cells in a fibrotic stroma. (b) Immunohistochemical analysis of the tumor revealed a loss of fumarate hydratase (FH) staining and (c) positive S‐(2‐succino)‐cysteine (2SC) staining. (d) The proband (indicated with the arrow head) was a 50‐year‐old man who was diagnosed with kidney cancer at the age of 48. His daughter, who also shared the *FH* variant, exhibited cutaneous and endometrial leiomyomas at age 29. His other daughter, who did not report clinical features associated with HLRCC, did not share the *FH* variant. Little information is available pertaining to the health of the patient's extended maternal and paternal relatives

A 48‐year‐old male without a reported significant medical history presented with right upper quadrant abdominal pain. CT of the abdomen revealed a 15‐cm complex cystic mass from the right kidney inferior pole, with liver and lytic bone lesions suspicious for metastasis. Biopsy of the left iliac bone lesion was performed and revealed small fragments of a poorly differentiated spindle and epithelioid neoplasm (Figure 2a). Immunostains showed the cells had patchy positivity for Pax‐8 and GATA‐3, and focal weak positivity for pancytokeratin and p63, while they were negative for TTF‐1, CK7, CK20, CA‐IX, and 34‐beta‐E‐12, compatible with a metastasis from a sarcomatoid neoplasm of renal origin. The patient initiated treatment under a clinical trial with an vascular endothelial growth factor (VEGF) inhibitor and checkpoint inhibitor.

NGS somatic analysis of the bone metastasis revealed the following alterations: KRAS (NM_033360) exon 2 p.G12D (c.35G > A) and NF2 (NM_000268) exon 9 p.K284* (c.850_854delAAGTT). Germline analysis of 76 cancer predisposition genes revealed the heterozygous c.914T > C (p.Phe305Ser) variant in the *FH*. Loss of heterozygosity for FH was detected in the tumor, and immunohistochemistry performed at this time showed loss of FH staining (Figure 2b) and positive 2SC staining (Figure 2c). The patient passed away 31 months after diagnosis from metastatic disease. His family history was significant for his father, who died in his 60s from cancer of unknown origin, although this diagnosis was not reported consistently among other family members, and his maternal grandfather who reportedly died from stomach cancer. Little information is available pertaining to the health of his extended relatives. The patient is of African American, Native American, and Irish descent (Figure 2d). He was referred to dermatology for evaluation of treatment‐related complaints. Complete cutaneous examination was performed without documentation of any cutaneous leiomyomas. His 29‐year‐old daughter underwent predictive testing and was also found to carry the c.914T > C (p.Phe305Ser) variant in the *FH*. She was noted to have cutaneous facial leiomyomas, although she did not undergo a formal dermatologic assessment. Her history is also significant for an enlarged leiomyomatous uterus which was confirmed by CT of the chest, abdomen, and pelvis. The proband also has a 25‐year‐old daughter who underwent predictive genetic testing and was not found to carry the familial *FH* variant. She denied a personal history of HLRCC‐associated clinical features.

## 4 CONCLUSION

Under an institutional protocol, NGS somatic and germline analyses of cancer‐associated genes are offered to patients with advanced cancer. Of the 13,722 advanced patients (including 560 with renal cell carcinoma) who have consented for somatic and germline analysis, we have identified only two individuals with the *FH* c.914T > C (p.Phe305Ser) variant. Strikingly, both of these individuals had FH‐deficient renal cell carcinoma. Interestingly, both of these individuals self‐reported African American and Native American ancestry and the one chromosome in gnomAD with this reported variant in an individual of African descent. Additional studies are needed to determine if this pathogenic germline variant is more common in individuals of any particular ethnicity. In Case #1, we suspect that the *FH* mutation is segregating with the patient's maternal family, given the confirmed diagnosis of his cousin's early onset kidney cancer at age 40 and given his mother's reported history of uterine leiomyomas. Although the patient reported a history of kidney cancer in his father, this diagnosis was unconfirmed. In Case #2, we demonstrate that the variant is segregating with disease in the proband's daughter who is affected with leiomyomas. While clear loss of function variants, such as nonsense, frameshift, splice‐site variants, and gene deletions, have been reported in *FH* in association with HLRCC and are relatively straightforward to classify as pathogenic, missense variants can pose a challenge to classification. However, in *FH*, the majority of pathogenic variants identified are actually missense, suggesting the importance of studying *FH* missense variants in detail to ensure accurate classification (Pithukpakorn, 2006).

To our knowledge, this *FH* c.914T > C (p.Phe305Ser) missense variant, which does not have a Clinvar entry (accessed 1/29/2020), has only been reported once in the literature. That case report was an individual with FH‐deficient renal cell carcinoma which was diagnosed before the age of 30 (Cajaiba et al., 2018). Here, we report this germline *FH* c.914T > C (p.Phe305Ser) variant in two additional immunohistochemistry and molecularly proven FH‐deficient renal cell carcinoma cases, and demonstrate that the variant segregates with disease in a proband's family. The *FH* c.914T > C (p.Phe305Ser) is classified as pathogenic for HLRCC.

## CONFLICT OF INTEREST

LZ’s family member has a leadership position and ownership interest of Shanghai Genome Center. The other authors have no conflict of interest.

## AUTHOR CONTRIBUTIONS

KB and DM were responsible for conceptualizing this study and drafting the manuscript. KB was responsible for organizing and analyzing patient data. YBC conducted and analyzed the functional studies. YK, AM, LZ, OCB, MC, KB, and DM performed data analysis and interpretation. All authors edited and approved the final draft.

## Data Availability

These data have not yet been deposited in a public database such as ClinVar.
